# Supplementary material for: A Likelihood Approach for Real-Time Calibration of Stochastic Compartmental Epidemic Models
Source: PLoS Comput Biol. 2017 Jan 17;13(1):e1005257. doi: 10.1371/journal.pcbi.1005257 (PMC5240920; doi:10.1371/journal.pcbi.1005257)
Supplement: S1 File — (TAR.GZ) [file pcbi.1005257.s014.tar.gz › HSPH_Online-SI-Revision/EnKF07/n10-severe/n10-severe_EnKF_table4.pdf]

1	R0	{1.68952, 11.8402}	{2.55134, 6.85516}	{{1.48399, 2.42537}, {3., 10.3922}}	{54.6563, 42.1027}	inf	584.	386.621	{170.142, 449.619}	47.4094	0.297224	newly	319.	453.86	{111., 624.}	93.3417	0.297156	Reff	0.653675	0.570508	{0.235479, 1.2459}	57.8273	ar	6872.	15 906.7	{14 507., 18 375.}	131.472
2	R0	{1.71739, 9.62661}	{2.06926, 4.85208}	{{0.731336, 0.598898}, {3., 6.4749}}	{67.1805, 55.0078}	inf	487.	460.851	{24.9129, 776.371}	109.081	0.647664	newly	307.	660.3	{20., 1827.}	204.632	0.616316	Reff	0.669954	0.415303	{0.137408, 0.702492}	50.5994	ar	6874.	16 038.2	{14 834., 17 712.}	133.317
3	R0	{1.48936, 6.23975}	{2.73781, 7.58093}	{{2.12602, 0.0193393}, {3., 16.5055}}	{83.8244, 89.1093}	inf	196.	827.183	{46.8803, 1046.95}	370.899	0.488334	newly	176.	1357.86	{26., 5202.}	736.148	0.703969	Reff	0.69285	0.599163	{0.208717, 0.929317}	52.1053	ar	5666.	17 810.4	{15 343., 25 409.}	214.338
4	R0	{1.62394, 7.51135}	{2.35044, 6.17498}	{{1.31671, 3.40029}, {3., 9.5886}}	{58.6407, 47.8863}	inf	314.	1295.7	{572.802, 1281.81}	316.1	0.482807	newly	272.	1296.79	{296., 2470.}	378.813	0.504794	Reff	0.684491	0.43208	{0.0680298, 0.822043}	50.1018	ar	6417.	16 962.6	{15 319., 20 290.}	164.339
5	R0	{1.53632, 8.57652}	{2.41632, 7.22899}	{{1.38044, 0.826877}, {3., 14.3199}}	{61.6861, 68.9229}	inf	366.	432.417	{30.5244, 371.19}	113.194	0.490352	newly	274.	406.46	{14., 595.}	123.314	0.500534	Reff	0.697643	0.593815	{0.258282, 1.34082}	55.6843	ar	6260.	15 465.6	{14 044., 19 586.}	147.054
6	R0	{1.47166, 5.7434}	{2.66237, 8.6091}	{{1.94875, 1.73115}, {3., 15.148}}	{80.9092, 116.867}	inf	206.	1114.96	{195.62, 784.367}	451.856	0.626771	newly	216.	827.77	{98., 1378.}	301.31	0.574952	Reff	0.704484	0.525837	{0.0115907, 1.20176}	60.6953	ar	5650.	17 079.5	{14 774., 22 352.}	202.291
7	R0	{1.48179, 8.87369}	{2.32837, 6.33225}	{{0.97228, 3.4249}, {3., 10.8796}}	{67.866, 46.2726}	inf	206.	754.44	{447.188, 670.622}	266.233	0.477433	newly	142.	796.01	{332., 955.}	460.57	0.5844	Reff	0.774828	0.402517	{0.0542986, 0.810749}	59.8141	ar	5233.	16 185.5	{14 997., 18 257.}	209.296
8	R0	{1.53669, 6.47807}	{2.55837, 5.50387}	{{1.57448, 3.55814}, {3., 7.57036}}	{66.4857, 30.9372}	inf	235.	578.384	{76.3939, 1281.43}	166.621	0.311799	newly	203.	539.92	{59., 1361.}	187.33	0.367829	Reff	0.682444	0.493146	{0.187146, 0.778653}	44.3587	ar	5934.	17 317.1	{15 561., 23 038.}	191.828
9	R0	{1.57227, 6.20989}	{2.62936, 11.7613}	{{2.0712, 6.44292}, {3., 15.6555}}	{67.2334, 89.3962}	inf	357.	206.756	{43.7094, 168.914}	93.3378	0.591043	newly	361.	120.71	{15., 121.}	89.8532	0.830558	Reff	0.71271	0.557651	{0.207998, 0.958382}	49.756	ar	6136.	16 096.8	{14 889., 18 004.}	162.334
10	R0	{1.54936, 6.50938}	{2.67222, 10.9482}	{{2.03594, 6.35428}, {3., 14.1918}}	{72.4724, 70.1449}	inf	238.	260.061	{46.5843, 254.408}	105.944	0.382698	newly	209.	157.52	{18., 141.}	98.5072	0.546205	Reff	0.623308	0.513401	{0.153649, 0.812592}	53.4844	ar	6275.	16 387.7	{15 165., 17 375.}	161.158
11	R0	{1.42179, 3.18601}	{2.56154, 12.3215}	{{1.67425, 1.12142}, {3., 19.0366}}	{80.1628, 334.531}	inf	53.	299.41	{17.6659, 332.667}	488.625	0.57756	newly	90.	301.86	{8., 667.}	289.489	0.571499	Reff	0.684876	0.562352	{0.194339, 0.943219}	48.4612	ar	5322.	16 102.2	{14 965., 16 710.}	202.558
12	R0	{1.48035, 6.46183}	{2.42959, 11.4785}	{{1.51463, 0.950979}, {3., 19.8215}}	{64.6825, 151.734}	inf	313.	249.186	{30.6933, 397.513}	53.1279	0.381529	newly	284.	242.44	{25., 595.}	86.8732	0.571748	Reff	0.695468	0.540046	{0.251332, 1.10469}	46.5279	ar	5793.	16 420.4	{15 336., 17 749.}	183.452
13	R0	{1.75755, 8.53854}	{2.42562, 5.64298}	{{1.52428, 3.59528}, {3., 8.29677}}	{48.2327, 33.9117}	inf	415.	728.782	{351.977, 869.716}	109.159	0.189718	newly	276.	610.44	{230., 1078.}	132.71	0.227644	Reff	0.648009	0.536752	{0.101581, 1.076}	46.8718	ar	6832.	17 706.	{15 867., 21 094.}	159.163
14	R0	{1.47355, 4.89451}	{2.23811, 5.71711}	{{0.728928, 3.56135}, {3., 6.59764}}	{84.8529, 32.6046}	inf	140.	292.3	{68.9469, 368.169}	128.128	0.221585	newly	163.	274.87	{66., 296.}	116.215	0.254323	Reff	0.670023	0.37028	{0.0955545, 0.756177}	52.7396	ar	5710.	15 877.8	{15 290., 15 855.}	178.069
15	R0	{1.62071, 5.98665}	{1.97389, 2.59596}	{{0.847241, 0.48597}, {3., 3.57863}}	{63.3125, 69.8473}	inf	218.	376.907	{148.552, 221.063}	133.255	0.250505	newly	197.	351.84	{137., 251.}	108.497	0.186602	Reff	0.578593	0.254228	{0.0764058, 0.456675}	60.9685	ar	6635.	17 390.1	{16 093., 18 680.}	162.096
16	R0	{1.67268, 7.7256}	{2.2091, 6.96339}	{{0.3718, 3.56614}, {3., 11.4667}}	{75.5291, 51.5116}	inf	248.	280.408	{31.8076, 421.885}	66.952	0.313449	newly	184.	298.96	{32., 512.}	128.38	0.384569	Reff	0.552988	0.418172	{0.0510572, 0.811219}	56.4307	ar	6947.	15 597.6	{14 872., 16 503.}	124.522
17	R0	{1.67805, 11.1417}	{2.0782, 7.34192}	{{0.88168, 3.54891}, {3., 11.6191}}	{65.962, 38.1322}	inf	598.	421.186	{146.705, 878.064}	55.7078	0.329018	newly	353.	844.16	{124., 2028.}	205.745	0.420826	Reff	0.775091	0.705919	{0.178111, 1.01587}	68.346	ar	6625.	17 373.5	{15 355., 23 003.}	162.241
18	R0	{1.66743, 9.99046}	{2.45876, 2.76748}	{{1.61964, 2.22623}, {3., 3.36139}}	{49.6365, 72.2988}	inf	590.	852.362	{46.7389, 532.876}	114.411	0.421079	newly	394.	1420.15	{66., 2509.}	298.013	0.468261	Reff	0.682146	0.64251	{0.204793, 1.21584}	41.1299	ar	6899.	17 683.5	{15 418., 21 881.}	156.32
19	R0	{1.70787, 9.85478}	{2.2734, 8.09345}	{{0.960838, 1.75114}, {3., 14.533}}	{56.1628, 56.4669}	inf	473.	531.506	{39.4937, 295.326}	108.134	0.506775	newly	313.	364.81	{18., 509.}	84.4569	0.470823	Reff	0.632595	0.535446	{0.0729858, 1.03347}	72.8648	ar	6867.	15 830.2	{14 209., 20 351.}	130.526
20	R0	{1.53809, 10.5848}	{2.68148, 10.463}	{{1.73193, 7.36491}, {3., 12.8396}}	{74.3382, 24.4018}	inf	613.	334.574	{27.7402, 366.479}	94.6417	0.749518	newly	373.	241.6	{14., 350.}	103.775	0.776623	Reff	0.804882	0.67084	{0.101478, 1.73077}	66.507	ar	6108.	15 566.2	{13 764., 21 005.}	154.849
21	R0	{1.59925, 6.74479}	{2.61382, 10.8709}	{{2.0098, 6.38783}, {3., 15.042}}	{63.4404, 66.2561}	inf	243.	351.082	{69.9037, 325.522}	141.378	0.422952	newly	202.	204.22	{34., 222.}	117.455	0.540803	Reff	0.649775	0.548966	{0.0662701, 1.4068}	67.9551	ar	6411.	17 011.6	{15 163., 23 725.}	165.351
22	R0	{1.38779, 4.40977}	{2.07736, 8.66637}	{{0.897142, 3.52777}, {3., 19.7966}}	{80.4231, 113.728}	inf	176.	498.594	{124.077, 895.08}	195.429	0.358254	newly	221.	737.43	{94., 1208.}	281.824	0.381832	Reff	0.751211	0.581411	{0.12443, 0.916341}	71.9061	ar	5154.	17 199.6	{15 329., 24 710.}	233.714
23	R0	{1.53657, 7.50352}	{2.67567, 11.3592}	{{2.14649, 7.00629}, {3., 16.94}}	{74.1324, 58.8072}	inf	330.	644.127	{39.8185, 426.378}	173.936	0.493755	newly	276.	505.83	{15., 513.}	180.272	0.650168	Reff	0.757222	0.508714	{0.0662659, 0.734639}	52.5426	ar	5928.	16 869.5	{15 080., 19 755.}	184.573
24	R0	{1.71131, 6.24526}	{2.10201, 7.37383}	{{0.836474, 3.58698}, {3., 11.4762}}	{65.1941, 62.3381}	inf	308.	500.921	{169.187, 677.49}	99.1864	0.236926	newly	276.	805.17	{108., 1039.}	252.873	0.39522	Reff	0.599814	0.581903	{0.0151907, 1.10296}	66.5935	ar	6924.	16 943.9	{15 213., 20 463.}	144.713
25	R0	{1.4412, 3.07729}	{2.47794, 5.74125}	{{0.0295649, 0.706577}, {3., 10.21}}	{97.5335, 158.653}	inf	56.	535.014	{61.7064, 511.602}	861.898	0.615233	newly	93.	690.92	{33., 672.}	684.602	0.599588	Reff	0.624328	0.386308	{0.00701866, 0.764221}	51.2454	ar	5810.	17 261.6	{16 075., 18 274.}	197.101
26	R0	{1.4159, 4.93858}	{2.69311, 10.953}	{{2.13683, 6.5002}, {3., 15.6339}}	{90.2046, 121.785}	inf	215.	410.615	{167.445, 441.163}	133.304	0.218892	newly	264.	232.06	{59., 248.}	91.3788	0.426131	Reff	0.740657	0.638747	{0.247326, 1.54666}	54.1375	ar	5255.	17 465.2	{15 726., 20 642.}	232.353
27	R0	{1.57465, 7.05414}	{2.07752, 6.28954}	{{0.223765, 0.553578}, {3., 10.6233}}	{88.246, 68.241}	inf	430.	730.743	{31.8673, 1730.54}	169.17	0.614779	newly	370.	865.45	{14., 2765.}	226.278	0.723671	Reff	0.69946	0.371224	{0.0318028, 0.679313}	68.3261	ar	6359.	17 900.2	{15 649., 21 377.}	181.493
28	R0	{1.41422, 4.62402}	{2.5936, 11.9797}	{{1.98399, 6.39551}, {3., 15.7378}}	{83.3942, 159.075}	inf	181.	244.719	{48.7326, 235.571}	120.245	0.363874	newly	219.	137.61	{22., 131.}	96.4703	0.648372	Reff	0.681654	0.511111	{0.132629, 0.851888}	51.8075	ar	5485.	16 064.2	{14 986., 16 329.}	192.874
29	R0	{1.41901, 3.63017}	{2.01692, 6.14234}	{{0.781685, 3.63155}, {3., 7.88713}}	{77.4623, 69.2025}	inf	109.	377.001	{237.534, 504.918}	253.69	0.457864	newly	157.	305.24	{139., 454.}	106.599	0.232863	Reff	0.594707	0.328498	{0.111174, 0.546871}	47.257	ar	5979.	17 815.3	{16 631., 20 380.}	197.965
30	R0	{1.60719, 4.72984}	{2.22711, 5.89169}	{{1.32597, 3.43997}, {3., 7.89765}}	{54.4542, 49.1082}	inf	159.	496.374	{111.619, 337.586}	228.215	0.325736	newly	176.	604.15	{119., 486.}	261.006	0.239256	Reff	0.595464	0.411298	{0.14943, 0.865291}	56.375	ar	6479.	18 166.4	{16 680., 22 183.}	180.388
31	R0	{1.38175, 6.26468}	{2.58843, 11.9605}	{{1.71333, 1.20012}, {3., 18.1192}}	{87.3298, 148.869}	inf	255.	270.412	{23.1688, 392.246}	84.6973	0.443031	newly	236.	373.84	{16., 753.}	157.703	0.647295	Reff	0.70939	0.602424	{0.176916, 1.42702}	61.9389	ar	5397.	16 579.9	{15 062., 21 666.}	207.205
32	R0	{1.4947, 8.8847}	{2.47374, 6.96858}	{{1.47677, 3.41916}, {3., 9.45109}}	{71.5907, 37.4891}	inf	294.	804.712	{526.322, 909.285}	175.249	0.348032	newly	208.	885.85	{276., 1217.}	327.88	0.407564	Reff	0.787707	0.427737	{0.0641593, 0.625055}	57.6963	ar	5407.	16 445.4	{15 169., 20 617.}	204.149
33	R0	{1.55891, 8.28978}	{2.37036, 4.29137}	{{1.31527, 3.17411}, {3., 4.32231}}	{58.8783, 52.3168}	inf	374.	752.66	{122.519, 1492.78}	150.633	0.291001	newly	292.	878.58	{98., 1862.}	238.13	0.330165	Reff	0.720061	0.378575	{0.135953, 0.569867}	57.5717	ar	6239.	18 069.5	{15 952., 22 541.}	189.621
34	R0	{1.60764, 10.4729}	{2.28943, 2.41336}	{{0.881858, 0.563219}, {3., 1.91333}}	{61.5214, 76.9561}	inf	407.	215.536	{116.935, 116.935}	83.4864	0.538205	newly	229.	300.37	{117., 534.}	87.6376	0.280678	Reff	0.67971	0.430636	{0.0548642, 1.06853}	63.0245	ar	6495.	15 267.	{14 369., 16 938.}	135.057
35	R0	{1.48527, 5.74485}	{2.61173, 11.5586}	{{1.66667, 1.00686}, {3., 19.2716}}	{75.8499, 170.159}	inf	235.	246.671	{61.9166, 395.182}	58.7097	0.308955	newly	239.	300.18	{26., 624.}	110.837	0.503008	Reff	0.703869	0.530459	{0.172428, 0.872847}	48.1449	ar	5811.	16 108.3	{15 179., 16 694.}	177.204
36	R0	{1.61457, 9.93364}	{2.17811, 6.35216}	{{0.903751, 0.973619}, {3., 9.84167}}	{67.8536, 36.0559}	inf	545.	884.651	{48.0033, 1690.98}	145.472	0.481161	newly	332.	639.79	{34., 855.}	150.708	0.465516	Reff	0.74577	0.431586	{0.061538, 0.736541}	48.1339	ar	6338.	17 240.9	{14 967., 23 285.}	172.024
37	R0	{1.47831, 7.08139}	{2.06295, 7.55007}	{{0.889099, 3.54459}, {3., 11.8893}}	{75.7388, 58.5613}	inf	340.	336.9	{99.8483, 698.702}	61.6793	0.296406	newly	278.	576.1	{86., 1112.}	182.525	0.451659	Reff	0.759556	0.620088	{0.179232, 0.947241}	58.714	ar	5530.	16 714.8	{15 249., 19 275.}	202.258
38	R0	{1.73445, 9.8858}	{2.1375, 7.25785}	{{0.851949, 3.56106}, {3., 11.5976}}	{65.7802, 42.5132}	inf	569.	529.802	{186.807, 774.643}	77.7747	0.343656	newly	367.	958.34	{133., 978.}	232.251	0.403029	Reff	0.688056	0.664409	{0.186298, 0.89822}	56.8442	ar	6842.	17 007.1	{15 238., 20 360.}	148.569
39	R0	{1.66621, 11.4824}	{2.81037, 9.66741}	{{2.352, 6.0147}, {3., 13.7071}}	{68.6685, 33.716}	inf	533.	382.072	{141.807, 330.017}	57.2526	0.29904	newly	292.	256.3	{65., 344.}	47.2877	0.254827	Reff	0.756459	0.579937	{0.272643, 0.748915}	45.7493	ar	6473.	16 135.3	{14 992., 19 921.}	149.27
40	R0	{1.60694, 9.95741}	{2.77561, 5.61988}	{{2.38199, 1.32879}, {3., 9.54235}}	{72.7264, 43.5873}	inf	510.	1092.38	{46.0365, 3548.51}	151.668	0.427845	newly	314.	1311.2	{33., 3809.}	343.65	0.528209	Reff	0.690181	0.578277	{0.174014, 1.07589}	57.3278	ar	6625.	17 572.1	{14 432., 23 179.}	165.24
41	R0	{1.55128, 4.71911}	{2.49669, 6.25339}	{{1.69338, 3.74998}, {3., 8.06621}}	{60.9441, 49.7626}	inf	233.	459.296	{107.619, 552.674}	120.091	0.19281	newly	259.	566.49	{122., 879.}	184.622	0.312194	Reff	0.655881	0.434134	{0.135925, 0.766315}	60.8418	ar	6141.	18 207.1	{16 630., 23 644.}	196.485
42	R0	{1.60063, 6.81118}	{2.66913, 8.54987}	{{2.01551, 4.07519}, {3., 14.652}}	{66.7551, 46.1965}	inf	295.	1502.56	{112.871, 4272.07}	434.943	0.626754	newly	240.	1147.87	{83., 3505.}	408.204	0.636747	Reff	0.619924	0.527197	{0.164773, 1.00618}	56.1796	ar	6528.	18 703.1	{15 289., 27 412.}	186.505
43	R0	{1.56986, 5.44652}	{1.96699, 6.05213}	{{0.268522, 0.478104}, {3., 10.262}}	{87.6669, 89.3664}	inf	202.	533.572	{43.6856, 436.689}	219.818	0.403998	newly	196.	586.29	{22., 1426.}	271.097	0.579477	Reff	0.625118	0.32143	{0.0182786, 0.916939}	67.0111	ar	6312.	17 363.1	{16 002., 18 817.}	175.08
44	R0	{1.47523, 8.07785}	{2.28886, 6.40289}	{{0.979508, 0.689766}, {3., 10.7918}}	{76.9942, 57.7576}	inf	323.	832.384	{141.847, 1465.31}	178.581	0.335544	newly	237.	731.94	{79., 929.}	232.068	0.436215	Reff	0.760776	0.423635	{0.0710676, 0.969034}	58.3916	ar	5692.	17 124.2	{15 111., 20 921.}	200.847
45	R0	{1.6417, 7.69429}	{2.05008, 6.87258}	{{0.797224, 3.58146}, {3., 8.56801}}	{71.1002, 25.6686}	inf	363.	293.225	{102.791, 453.708}	72.079	0.366988	newly	273.	382.02	{85., 556.}	127.971	0.398853	Reff	0.605951	0.476871	{0.0720285, 0.794955}	48.9908	ar	6735.	16 130.4	{15 170., 17 233.}	139.501
46	R0	{1.38865, 5.83473}	{2.7078, 3.93412}	{{2.18835, 3.86847}, {3., 4.10672}}	{94.9955, 32.574}	inf	305.	739.694	{55.1961, 1496.13}	193.211	0.434627	newly	319.	1451.02	{61., 4702.}	398.47	0.511757	Reff	0.760702	0.829273	{0.109034, 2.18813}	84.2162	ar	5233.	18 004.	{15 376., 25 795.}	244.048
47	R0	{1.45289, 6.87903}	{2.15317, 7.40053}	{{0.880549, 3.53429}, {3., 11.589}}	{79.5832, 58.1482}	inf	301.	315.02	{52.9623, 674.996}	63.5462	0.32795	newly	259.	549.54	{78., 1357.}	188.903	0.474583	Reff	0.779475	0.65858	{0.167752, 0.938766}	56.218	ar	5379.	16 681.9	{15 153., 20 773.}	210.13
48	R0	{1.69416, 9.4831}	{2.28667, 10.533}	{{1.01161, 7.03042}, {3., 14.2255}}	{62.068, 37.4524}	inf	573.	610.403	{154.299, 1094.84}	73.5411	0.306006	newly	377.	376.73	{75., 734.}	81.7374	0.38833	Reff	0.683255	0.384568	{0.0726831, 0.570365}	54.1775	ar	6925.	16 940.2	{15 612., 20 176.}	144.624
49	R0	{1.47843, 7.16286}	{2.67773, 6.94066}	{{2.15193, 0.538902}, {3., 15.7706}}	{81.1201, 103.705}	inf	252.	814.96	{38.188, 1195.22}	264.557	0.498391	newly	214.	1545.93	{21., 6965.}	670.575	0.687622	Reff	0.752669	0.631411	{0.167266, 1.38152}	57.2887	ar	5449.	17 673.6	{15 415., 23 723.}	224.346
50	R0	{1.68843, 5.72427}	{1.97383, 6.87625}	{{0.626036, 1.3921}, {3., 15.0029}}	{69.0985, 99.2309}	inf	170.	795.687	{84.9369, 411.939}	388.442	0.524562	newly	156.	748.93	{48., 662.}	407.365	0.50812	Reff	0.581326	0.273728	{0.0520327, 0.581037}	66.6544	ar	6800.	17 930.1	{16 318., 20 828.}	163.678
